# Transmission of a Novel Imprinting Center Deletion Associated With Prader–Willi Syndrome Through Three Generations of a Chinese Family: Case Presentation, Differential Diagnosis, and a Lesson Worth Thinking About

**DOI:** 10.3389/fgene.2021.630650

**Published:** 2021-08-24

**Authors:** Kaihui Zhang, Shu Liu, Wenjun Gu, Yuqiang Lv, Haihua Yu, Min Gao, Dong Wang, Jianyuan Zhao, Xiaoying Li, Zhongtao Gai, Shimin Zhao, Yi Liu, Yiyuan Yuan

**Affiliations:** ^1^Obstetrics and Gynecology Hospital of Fudan University, Fudan University, Shanghai, China; ^2^Pediatric Research Institute, Qilu Children’s Hospital of Shandong University, Jinan, China; ^3^State Key Laboratory of Genetic Engineering and School of Life Sciences, Fudan University, Shanghai, China; ^4^Children Inherited Metabolism and Endocrine Department, Guangdong Women and Children Hospital, Guangzhou, China; ^5^Neonatal Intensive Care Unit, Qilu Children’s Hospital of Shandong University, Jinan, China; ^6^Key Laboratory of Reproduction Regulation of NPFPC, Collaborative Innovation Center of Genetics and Development, Institutes of Biomedical Sciences, Fudan University, Shanghai, China

**Keywords:** Prader–Willi syndrome, Prader–Willi-like syndrome, imprinting center, microdeletion, familial transmission

## Abstract

Prader–Willi syndrome (PWS) is a complex genetic syndrome caused by the loss of function of genes in 15q11-q13 that are subject to regulation by genomic imprinting and expressed from the paternal allele only. The main clinical features of PWS patients are hypotonia during the neonatal and infantile stages, accompanied by delayed neuropsychomotor development, hyperphagia, obesity, hypogonadism, short stature, small hands and feet, mental disabilities, and behavioral problems. However, PWS has a clinical overlap with other disorders, especially those with other gene variations or chromosomal imbalances but sharing part of the similar clinical manifestations with PWS, which are sometimes referred to as Prader–Willi syndrome-like (PWS-like) disorders. Furthermore, it is worth mentioning that significant obesity as a consequence of hyperphagia in PWS usually develops between the ages of 1 and 6 years, which makes early diagnosis difficult. Thus, PWS is often not clinically recognized in infants and, on the other hand, may be wrongly suspected in obese and intellectually disabled patients. Therefore, an accurate investigation is necessary to differentiate classical PWS from PWS-like phenotypes, which is imperative for further treatment. For PWS, it is usually sporadic, and very rare family history and affected siblings have been described. Here, we report the clinical and molecular findings in a three-generation family with a novel 550-kb microdeletion affecting the chromosome 15 imprinting center (IC). Overall, the present study finds that the symptoms of our patient are somewhat different from those of typical PWS cases diagnosed and given treatment in our hospital. The familial occurrence and clinical features were challenging to our diagnostic strategy. The microdeletion included a region within the complex small nuclear ribonucleoprotein polypeptide protein N (*SNRPN*) gene locus encompassing the PWS IC and was identified by using a variety of techniques. Haplotype studies suggest that the IC microdeletion was vertically transmitted from an unaffected paternal grandmother to an unaffected father and then caused PWS in two sibling grandchildren when the IC microdeletion was inherited paternally. Based on the results of our study, preimplantation genetic diagnosis (PGD) was applied successfully to exclude imprinting deficiency in preimplantation embryos before transfer into the mother’s uterus. Our study may be especially instructive regarding accurate diagnosis, differential diagnosis, genetic counseling, and PGD for familial PWS patients.

## Introduction

The Prader–Willi syndrome (PWS) is characterized by hypotonia and feeding problems in early infancy, as well as hypogonadism, small hands and feet, craniofacial signs, and hyperphagia leading to profound obesity. It is triggered by the loss of function of genes in 15q11-q13 that are regulated by genomic imprinting and expressed from the paternal allele only. In approximately 70% of the cases, PWS is the result of deletion of 5–7 Mb in the paternal 15q11-13 region; nearly 28% attributes to maternal uniparental disomy (mUPD); and in < 2%, it is developed as the consequence of mutation, microdeletion, or translocation disrupting the imprinting center (IC) (Buiting et al., 1995). In PWS IC microdeletion cases, the smallest region of microdeletion overlap (IC PWS-SRO) is about 4.3 kb and spans the promoter and exon 1 region of small nuclear ribonucleoprotein polypeptide N (SNRPN), and this region appears to be necessary for erasure of the maternal imprint and establishment and maintenance of the paternal imprint (Ohta et al., 1999). The vast majority of PWS patients, typically manifested as sporadic cases, are characterized by a low recurrence risk, whereas for PWS resulting from a familial microdeletion in the IC carried by the father, the recurrence risk could be as high as 50%. To the best of our knowledge, familial transmission of the IC microdeletion or multiple affected siblings have been proven to be very rare, and only four familial PWS cases with IC microdeletion transmitted through three generations have been reported so far (Hartin et al., 2018). This finding suggests that in families with an IC microdeletion, several generations may be unaffected and asymptomatic before some individual develops PWS. Even more complicated is that, recently, research has shown that phenotypic features typical of PWS can also be caused by other genetic variations that are associated with disorders defined as the so-called Prader–Willi syndrome-like (PWS-like) disorders (Gillessen-Kaesbach and Horsthemke, 1994; Lukusa and Fryns, 2000; Stalker et al., 2003; D’Angelo et al., 2006; Bonaglia et al., 2008a; Hosoki et al., 2009; Izumi et al., 2013; Schaaf et al., 2013; Desch et al., 2015; Dello Russo et al., 2016; Fountain et al., 2016; Geets et al., 2016; Linhares et al., 2016; Berger et al., 2017; McCarthy et al., 2018; Negishi et al., 2019). As a result, attempting to make a definite diagnosis of PWS as well as the differential diagnosing has become extremely challenging. Here, we reported two neonatal siblings with atypical but may be more severe phenotype of PWS triggered by a microdeletion in the PWS IC that was transmitted through three generations—unaffected paternal grandmother, unaffected father, and then these two affected sibling grandchildren. In the present study, we aimed to share our case presentation and explore some useful methodology for detecting the silent transmission of PWS IC microdeletion through the female germline that may cause considerable difficulties in diagnostic testing and genetic counseling in affected families.

## Materials and Methods

### Patients

**FIGURE 1:**
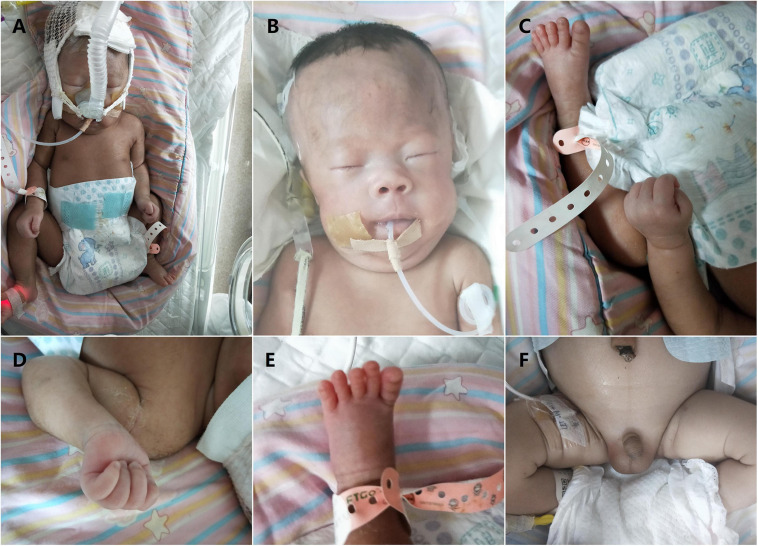
Clinical features of our patient with Prader–Willi syndrome (PWS) (2 days after birth). **(A)** Non-invasive respiratory support with positive end expiratory pressure was used for the treatment of respiratory distress. Overall appearance of the boy indicated no significant difference between the patient and normal infants. Note muscular hypotonia and abnormal position of the hands and feet. **(B)** Facial features of the boy (narrow forehead, fair eyebrow, bilateral epicanthus, flat nose, big nostrils, and thin upper lip). **(C)** Overall appearance of abnormal position of the hands and feet. **(D)** Abnormal position of the fingers with thumbs adducted under index and middle fingers, flexed hands and wrists, persistently clenched hands and arachnodactyly. **(E)** Small feet and toes. **(F)** Genital hypoplasia with slight maldescent of the left testis.

The 1-day-old male proposita was the second child of healthy non-consanguineous Chinese parents. Parental ages at birth of the child were 31 years (father) and 30 years (mother). During the pregnancy, ultrasound screening did not reveal any structural abnormalities except for decreased fetal activity. The first child of the couple was a baby girl who died 4 days after birth for unknown causes. Considering the family history of unexplained neonatal death, interventional prenatal diagnosis was recommended by the genetic counselor but refused by the couple. At 40 weeks + 5 days’ gestation, the patient was hospitalized with fetal distress. An elective cesarean section was performed on the day of admission due to breech presentation. The neonatal evaluation recorded 2,950 g in birth weight (17.8th centile), 50 cm in length (41.7th centile), and 35 cm in head circumference (65.6th centile), with an Apgar score of 10, 7, and 5 at 1, 5, and 10 min, respectively. The boy was transferred to the neonatal intensive care unit directly after birth because of severe respiratory distress, poor suck, and hypotonia. A thin male baby with poor response to external stimuli was noticed from a first glimpse at the patient. Physical examination revealed weak cry, flat nose, big nostrils, bilateral epicanthus, wide-spaced nipples, long slender fingers, and slight maldescent of the left testis (Figure 1). Neurological exam showed significant hypotonia. Ventilator and gastric tube feeding were applied on account of dyspnea and poor sucking. The boy died from respiratory infections (neonatal pneumonia), hypoventilation, and respiratory distress at the age of 10 days. Since the first child of the couple (the elder sister of the proposita) died 4 days after birth, retrospective analysis was conducted to compare the boy with his elder sister, with the results indicating more severe manifestation and rapid progression, particularly dyspnea and feeding difficulties, in the baby girl. In addition, as no similar findings were observed or reported from any of the other family members, neonatal lethal monogenic disorders such as fatty acid oxidation disorders and urea cycle disorder, as well as organic acid metabolism diseases including mitochondrial disease and chromosomal disease, were regarded as the overriding considerations. Also, PWS and PWS-like disorders could not be ruled out due to part of the clinical characteristics shared by the two siblings.

### Genetic Testing

#### Karyotyping

Peripheral blood samples from the patient and his family members were obtained for karyotyping, with their information anonymized prior to submission. Standard G-banded chromosome analysis at a 550-band resolution was performed using phytohemagglutinin (PHA)-stimulated peripheral blood lymphocytes prepared from the samples according to standard procedures. Chromosomal abnormalities were designated based on the International System for Human Cytogenetic Nomenclature guidelines (ISCN 2016).

#### DNA Collection and Extraction

To isolate genomic DNA, the QIAamp DNA blood mini kit (cat. no. 51104; Qiagen GmbH) was used according to the manufacturer’s protocol.

#### Whole-Exome Sequencing and Variant Calling

Proband DNA was sequenced to identify the causal gene. The DNA was isolated from peripheral blood with CWE9600 Automated Nucleic Acid Extraction System using CWE2100 Blood DNA Kit V2 (CWBiotech, China, CW2553). Here, 750 ng of genomic DNA was fragmented into 200–300 bp by Scientz08-III Ultrasonic Homogenizer (SCIENTZ, China). The DNA fragments were then processed by end-repairing, A-tailing, and adaptor ligation using KAPA Library Preparation Kit (Illumina, KR0453, v3.13), followed by an eight-cycle pre-capture PCR amplification. Then, the amplified DNA sample was captured in the Agilent SureSelect XT2 Target Enrichment System (Agilent Technologies, Inc., United States), with the captured DNA fragments purified by Dynabeads MyOne Streptavidin T1 (Invitrogen, Thermo Fisher Scientific, United States) and amplified by 13 cycles of post-capture PCR. The final products were further purified by Agencourt AMPure XP (Beckman Coulter, Inc., United States) and quantitated with Life Invitrogen Qubit 3.0 using Qubit dsDNA HS Assay Kit (Invitrogen, Thermo Fisher Scientific, United States). Eventually, quantified DNA was sequenced with 150-bp paired-end reads on Illumina Novaseq 6000 platform (Illumina, Inc., United States) according to the standard manual. The coverage contained the exon regions and adjacent intron regions (50 bp) of all human genes by SeqCap EZ Choice XL Library (Roche NimbleGen). The average sequencing depth of the target region for the proband was 123.02×, among which 96.49% of the target sequence had a sequencing depth of more than 20×. The father was 140.85×, of which 97.90% of the target sequence had a sequencing depth of more than 20×. The mother was 160.89×, of which 97.83% of the target sequence had a sequencing depth of more than 20×.

The raw data produced on Novaseq platform were filtered and aligned against the human reference genome (hg19) using Burrows–Wheeler Aligner (BWA)^1^ after being evaluated with Illumina Sequence Control Software (SCS). The single-nucleotide polymorphisms (SNPs) were called by using GATK software (Genome Analysis ToolKit)^2^. Variants were annotated using ANNOVAR^3^, and the effects of single-nucleotide variants (SNVs) were predicted by SIFT, Polyphen-2, and Mutation_Taster programs (Li J. et al., 2019; Li Z. et al., 2019). All variants were interpreted according to ACMG standards and then categorized to be pathogenic, likely pathogenic, variants of unknown clinical significance (VUS), likely benign, and benign.

#### Copy Number Variation Calls by Whole-Genome Sequencing

Here, 750 ng of genomic DNA was fragmented to an average size of 200–300 bp, and DNA libraries were constructed using KAPA Library Preparation Kit, with reagent employed for one of the libraries. The constructed DNA library samples were then taken for high-throughput sequencing with Illumina Nova Seq 6000. The sequencing depth is 0.6×, whole-genome low-depth sequencing. High-quality double-ended sequencing reads were aligned to the human reference genome sequence from the UCSC database using the BWA tool. The window width was preset at 50 Kb, with an adjustment amount of 5 Kb. A two-step calibration of guanine-cytosine (GC) and population model was performed across each of the windows. After removing the abnormal windows, the standard deviation between the copy ratio and the reference set of each window was calculated. A standard deviation of less than 0.15 determined by the software was considered to be in accordance with the quality control. The size and copy ratio of the final copy number variation (CNV) segments were calculated by identifying the break point. Afterward, identified and mapped CNVs were interrogated against publicly available databases, including Decipher, Database of Genomic Variants (DGV), 1,000 Genomes, and Online Mendelian Inheritance in Man (OMIM).

#### Chromosome Microarray Analysis

DNA from the patient, his father, and paternal grandmother was genotyped using InfiniumOmniZhongHua-8 array (Illumina, San Diego, CA, United States). In addition to a genome-wide functional resolution of approximately 20 kb for deletions and 50 kb for duplications, the array also had a higher density coverage of the 15q11-q13 region. The experiments were carried out under the manufacturer’s instructions. Genotype calling, quality control, and identification of CNV were performed using Illumina KaryoStudio software and cnvPartition algorithm, with various databases employed for array data evaluation and genotype–phenotype correlation analysis, including OMIM^4^, DECIPHER^5^, DGV^6^, and ISCA^7^.

#### Methylation-Specific Multiplex Ligation-Dependent Probe Amplification

Methylation-Specific Multiplex Ligation-dependent Probe Amplification (MS-MLPA^®^) reagents and kits obtained from MRC-Holland (MRC, Amsterdam, Netherlands) were used to verify the methylation status of chromosomes 15, including ME028-B2 kit containing sequence-specific probes that was applied for testing along the length of the 15q11.2-q13 region for the patient, his father, and paternal grandmother. In the presence of methylation-sensitive restriction enzymes, the B2 kit equipped with 48 MLPA probes was employed for copy number detection and methylation status verification. Approximately 50 ng of genomic DNA was introduced for each of the MS-MLPA reactions according to manufacturer’s instructions. The PCR products were analyzed by capillary electrophoresis on an ABI 3100 sequencer (Applied Biosystems, CA, United States) using GeneScan 500 LIZ dye Size Standard and formamide (Applied Biosystems, CA, United States), and GeneMarker version 2.64 (SoftGenetics, LLC) was used to determine the copy number and methylation status associated with the critical region of Prader–Willi syndrome/Angelman syndrome (PWS/AS) patients. The fluorescent signals from the copy number probes, by comparing with the normal controls, showed the ratios of 0.5 for deletions and 1.5 for duplications. Since the methylation probes were maternally imprinted (maternal allele methylated), the ratio of methylated probes to normal controls would increase accordingly in the presence of additional copies from maternal alleles but not paternal alleles.

## Results

### Karyotyping

All cases presented normal karyotypes (not presented).

#### Whole-Exome Sequencing and Copy Number Variation

**FIGURE 2:**
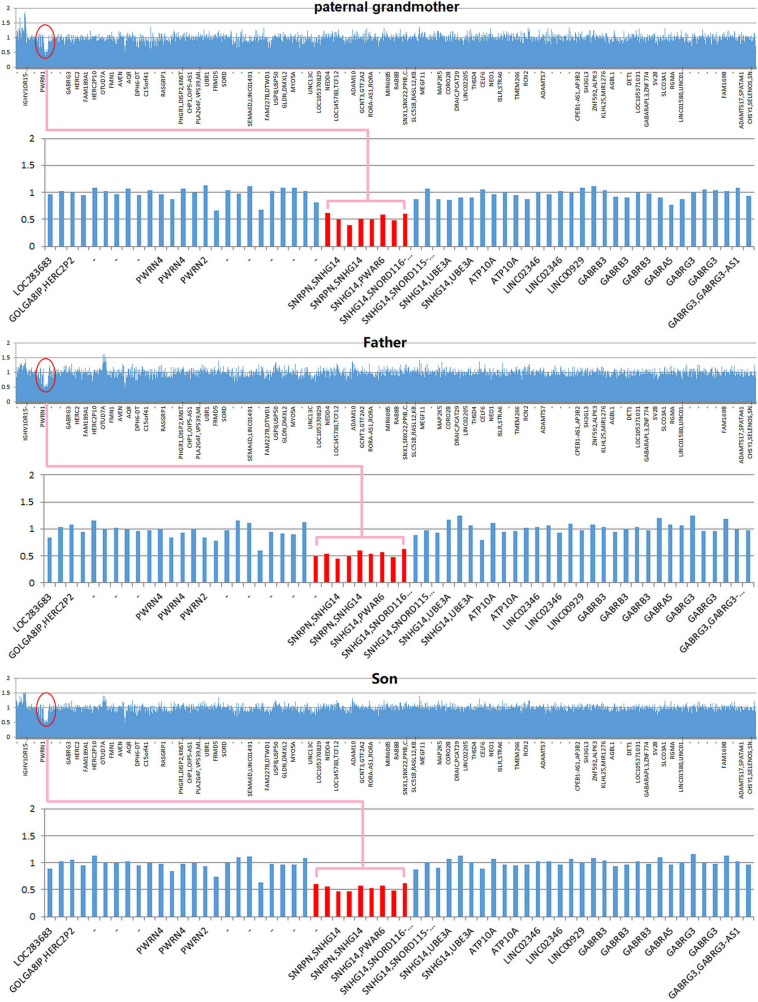
Copy number variation (CNV) sequencing reveals a *de novo* 500-kb heterozygous deletion of 15q11.2 (chr15: 24,932,524–25,482,598) in the paternal grandmother, the father, and the patient. The deletion encompasses five Online Mendelian Inheritance in Man (OMIM) genes including *SNRPN*, *SNHG14*, *PWAR6*, *SNORD115*, and *SNORD116* (highlighted in pink box).

Copy number variation analysis of the proband identified a 500-kb interstitial microdeletion of 15q11.2 with the first breakpoint located at 24,932,524 bp and the last breakpoint at 25,482,598 bp on the distal region. There are five genes reported in OMIM (*SNRPN*, *SNHG14*, *PWAR6*, *SNORD115*, and *SNORD116*) according to UCSC Genome Browser on Human February 2009 Assembly. We extended the genetic analysis to the family members of the patient (father, mother, and paternal grandmother). The same deletion was detected in the paternal grandmother and the father of the patient but not in the mother, supporting the paternal origin of the deletion (Figure 2).

Whole-exome sequencing (WES) did not show findings of variants with pathogenic significance in clinically relevant candidate genes causing PWS phenotype in this patient and other family members (Supplementary Table 1).

Since all of the other family members are in good health, the results suggested vertical transmission of the IC microdeletion from the unaffected paternal grandmother to the unaffected father, which consequently resulted in PWS in these two sibling grandchildren when the IC microdeletion was inherited paternally.

#### Methylation-Specific Multiplex Ligation-Dependent Probe Amplification

**FIGURE 3:**
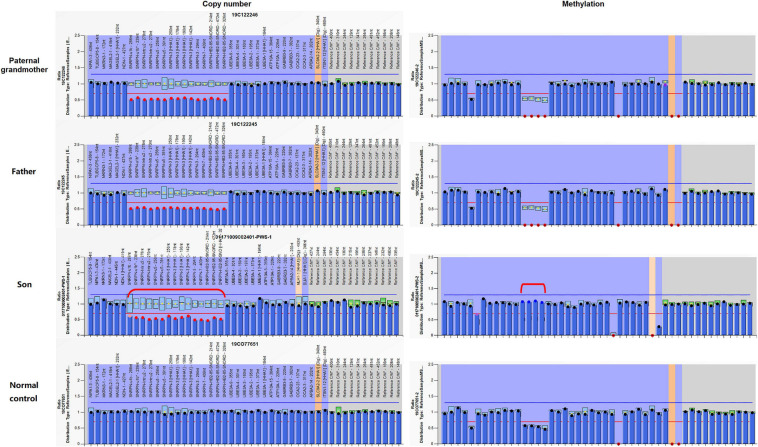
Copy number and methylation patterns generated using Methylation-Specific Multiplex Ligation-dependent Probe Amplification B2 kit (MS-MLPA-B2). The Prader–Willi syndrome/Angelman syndrome (PWS/AS) kit contains 48 probes for copy number detection and methylation status analysis that are specific to regions in or near the PWS/AS critical region on chromosome 15q11-q13. The left and right columns display the results of copy number and methylation pattern, respectively. In the left column, copy number peak ratios are determined by comparing patient with normal control (2 copies/2 copies = 1.0). The figure reveals deletion (showing a copy number of 1) in 15 probes in and around the *SNRPN* region (highlighted in red dots) in the paternal grandmother, the father, and the patient. Meanwhile, the normal control has a normal copy number of 2 for all analyzed gene fragments of chromosome 15. The methylation probes were designed to hybridize to maternally imprinted loci, shown in the right column. When compared to the normal control, each of the four probes within the deleted region has a ratio of around 0.5. In this family, the paternal grandmother and the father display an abnormal methylation pattern (ratio = 0) in the *SNRPN* region due to complete loss on the maternal chromosome 15, while the patient displays the typical PWS methylation pattern (ratio = 1) in the four *SNRPN* probes due to complete loss on his paternal chromosome 15. *UBE3A* exon 1 and one other digestion control probe were used during the methylation analysis. These results indicate that in the paternal grandmother and the father, the paternal allele is present, and the deletion is maternal in origin, which explains their absence of clinical symptoms. In contrast, the child displays an abnormal methylation pattern in the four methylation-sensitive fragments digested in the *SNRPN* region due to loss on his paternal chromosome 15.

Analysis of the patient and other family members was performed using an MS-MLPA kit specific for 15q11-q13 genomic region. Copy number changes were detected in 15 probes in the patient, indicating complete deletion of the *SNRPN* gene. The same deletion was also verified in the father and the paternal grandmother, but negative in the mother, which was corresponding to the paternal origin of the deletion as well. Methylation patterns within this region of this family revealed that the paternal grandmother and the father displayed an abnormal hypomethylation pattern in the *SNRPN* region due to complete loss on the maternal chromosome 15, whereas the patient presented with the typical PWS hypermethylation pattern in four *SNRPN* probes as a result of complete loss on his paternal chromosome 15 (Figure 3). In addition, a normal methylation pattern was observed in the mother. The findings from MS-MLPA confirmed the deletion of *SNURF/SNRPN* exon 1 and further identified that this deletion was paternal in origin.

#### Chromosome Microarray Analysis

**FIGURE 4:**
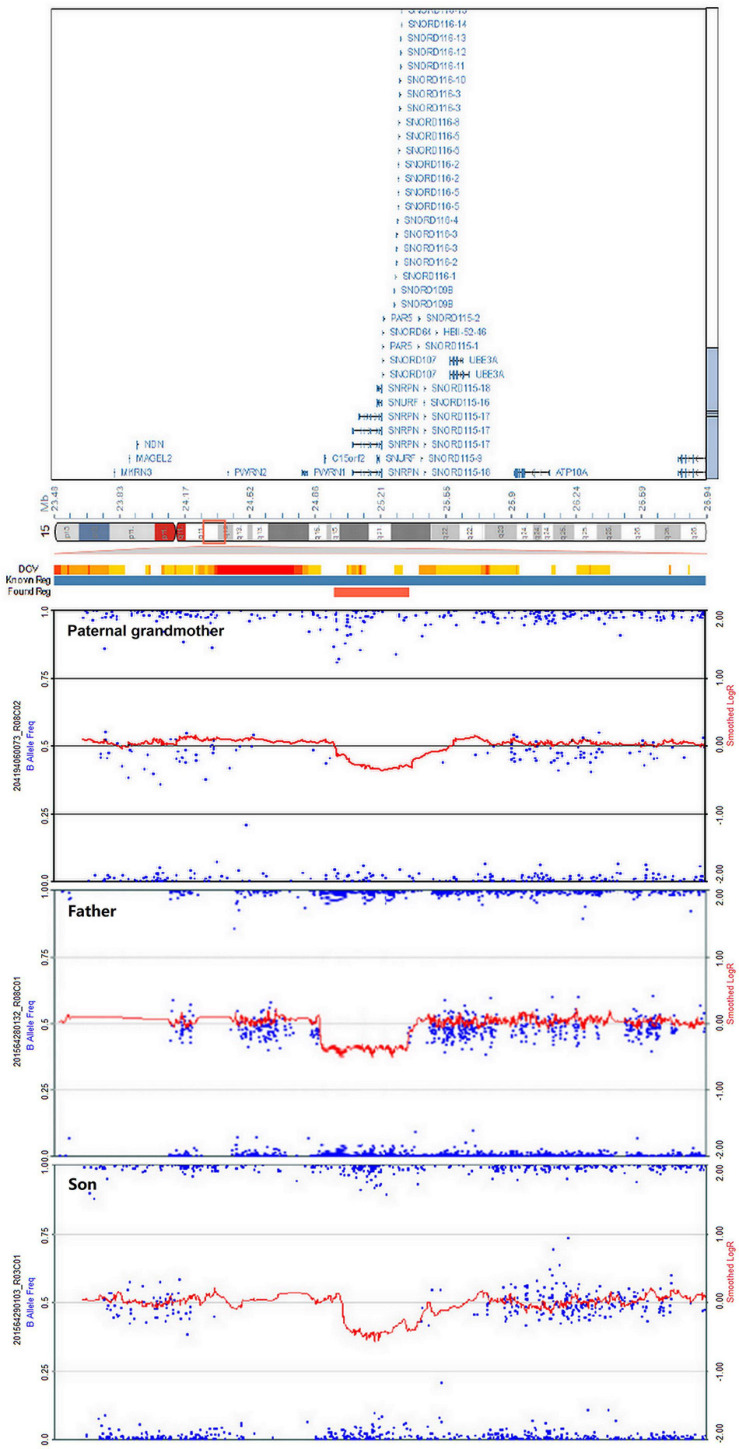
Chromosome microarray analysis (CMA) demonstrating deletion of 15q11.2 in the paternal grandmother, the father, and the patient. The novel copy number variation (CNV) at the loci (chr15: 24,966,348–25,364,551) is of approximately 398 kb in heterozygous state, surrounding the *SNRPN* gene.

High-resolution microarray analysis of this patient and other family members confirmed the results from CNV analysis data that demonstrated an interstitial microdeletion of 15q11.2. Meanwhile, trio analysis of SNP loci on chromosome 15 of the patient and his parent was also in accordance with the paternal inheritance. Chromosome microarray analysis (CMA) mapping revealed a 398-kb region of the chromosome 15 microdeletion, with the proximal breakpoint at 24,966,348 bp and the distal one at 25,364,551 bp (Figure 4). No additional aberrations were detected. As is known, *SNRPN*, *SNURF*, *PWRN2*, *SNORD116*, and *OCA2* are considered pathogenetic in the OMIM database. This deletion overlapped with upstream exons of the *SNURF-SNRPN* gene, thus verifying the findings from the MS-MLPA test.

In view of the clinical manifestation, karyotype, WES, CNV, CMA, and MS-MLPA, a diagnosis of PWS resulting from ∼550-kb loss on his paternal chromosome 15, which the breakpoint refers to the CNV calling for WGS, can eventually be confirmed for this patient [the sequencing reads for variant calling and the data for CMA had been deposited with NODE Bioproject (OEP001280 and OEP001281)]. The microdeletion of PWS IC was transmitted silently through two generations prior to being expressed in the third generation *via* the female germline—the paternal grandmother, the father, and then the two affected sibling grandchildren. With the aid of these genetic tools, PGD was therefore applied successfully to exclude imprinting deficiency in preimplantation embryos before transfer into the mother’s uterus (Reproductive Hospital of Shandong University). As expected, the mother has given birth to a healthy boy.

## Discussion

Prader–Willi syndrome is typically featured by hypotonia during the neonatal and infantile stage, accompanied by delayed neuropsychomotor development, hyperphagia, obesity, hypogonadism, short stature, small hands and feet, as well as mental disabilities and behavioral problems (Cassidy and Driscoll, 2009; Cassidy et al., 2012). However, it is not uncommon that its clinical phenotype may be confounded by disorders caused by other genetic variations, which were defined as PWS-like, and may present with similar manifestations (Rocha and Paiva, 2014; Cheon, 2016). Though PWS-like disorders share features of PWS phenotype, the genetic basis of these rare disorders differs. As is well known, PWS is usually triggered by a paternal deletion, maternal uniparental disomy, or imprinting defect of the chromosome region 15q11-q13, while the genetic etiologies of PWS-like disorders are more diverse, including variations in *MAGEL2* gene (Schaaf-Yang syndrome) (Schaaf et al., 2013) and *RAI1* gene (Smith–Magenis syndrome) (Alaimo et al., 2015), 1p36 monosomy (Tsuyusaki et al., 2010), 2pter deletion (Doco-Fenzy et al., 2014), deletion of 3p26.3 (Geuns et al., 2003), deletion of 6q (Bonaglia et al., 2008b; Izumi et al., 2013), 10q26 deletion (Lukusa and Fryns, 2000), 12q subtelomere deletions (Niyazov et al., 2007), chromosome 14 maternal uniparental disomy (Hosoki et al., 2009), paracentric inversion (X)(q26q28) (Florez et al., 2003), and duplication of X(q21.1-q21.31) (Pramyothin et al., 2010). Therefore, the variation of these genes in PWS-like disorders, though different from those of PWS, may be associated with phenotypes that are difficult to be differentiated from PWS due to the clinical overlap, especially atypical PWS, thus could consequently challenge the final diagnosis. The challenge for clinicians exists not only in accurate differentiation of the clinical manifestations between PWS and PWS-like disorders but also in the effort to provide conclusive genetic explanations for the phenotypes in order to offer uncompromised genetic counseling and treatment. Absence of correct diagnosis is highly likely to worsen the prognosis of the individuals due to the endocrine–metabolic malfunctioning associated with PWS. Given the phenotypic overlap between PWS and PWS-like disorders, tests aiming at other genetic variations should be considered in the context of PWS-like phenotypes but with negative results from PWS methylation analysis. Therefore, an appropriate and accurate genetic investigation strategy would be necessary and indispensable.

As a sporadic genetic disorder with remarkable developmental consequences, PWS is usually triggered by a paternal deletion, maternal uniparental disomy, or imprinting defect of the chromosome region 15q11-q13, which could be diagnosed using the standard methylation tests (MS-MLPA) with higher accuracy. The current genetic epidemiology indicates that approximately 65–75% PWS cases have a detectable deletion in this region, 20–30% cases are caused by mUPD, and imprinting errors have been observed in 1% (among 15% of the cases of either a sporadic or inherited microdeletion in the IC, there was a paternal chromosomal translocation in less than 1%) (Amos-Landgraf et al., 1999; Chai et al., 2003; Cassidy and Driscoll, 2009; Cassidy et al., 2012). Since the PWS patients with imprinting defect were rarely reported, no phenotypic feature is known to correlate exclusively with any of the three major molecular mechanisms that result in PWS. Previous research merely focused on the statistical differences in the frequency or severity of certain features between the two largest molecular classes (deletion and mUPD). To the best of our knowledge, comparison among the three classes did reveal discrepancies in the phenotype, typically demonstrated as less features in IC PWS cases, including decreased fetal movement, typical facial phenotypes, excessive or rapid weight gain, hyperphagia, hypopigmentation, small hands/feet, and thick saliva (Hartin et al., 2018). Moreover, imprinting center deletions can be inherited, result in an increased risk of recurrence, and therefore it is important to diagnose them in a timely matter to enable preconception counseling or PGD in families carrying this type of genetic anomaly.

In our research, we described a rare familial occurrence and atypical clinical features of PWS with a 550-kb microdeletion at 15q11.2: a typical IC deletion spanning *SNRPN*, *SNHG14*, *PWAR6*, *SNORD115*, and *SNORD116* on the maternal chromosome 15 of the paternal grandmother and the father. The IC deletion caused atypical PWS-like phenotype in the proposita and his elder sister. The microdeletion was transmitted silently through the female germline (the paternal grandmother and the father) but impaired the erasure of the maternal imprint and/or the establishment of a paternal imprint in the male germline (the proposita). So, the proposita inherited a paternal chromosome with a missing paternal imprint, leading to the development of PWS. The proposita and his elder sister exhibited part of the major clinical manifestations of PWS, including neonatal and infantile hypotonia, weak cry, feeding difficulties, and hypoplastic external genitalia, followed by recurrent respiratory infections. Interestingly, additional features such as low birth weight, characteristic facial features (narrow forehead, almond-shaped eyes, thin upper lip, downturned corner of the mouth), and hypopigmentation (fair skin and hair) that are common clinical features of PWS were not seen in this patient. These findings, accompanied by early neonatal death, once misled us to the consideration of neonatal lethal monogenic diseases (such as fatty acid oxidation disorders, urea cycle disorder, and organic acid metabolism disease), mitochondrial diseases, or chromosomal diseases in the first place. Therefore, the familial history of neonatal death and atypical clinical features, though presented as rare events in PWS patients, could lead clinical judgment astray, thus should arouse vigilance among pediatricians.

**FIGURE 5:**
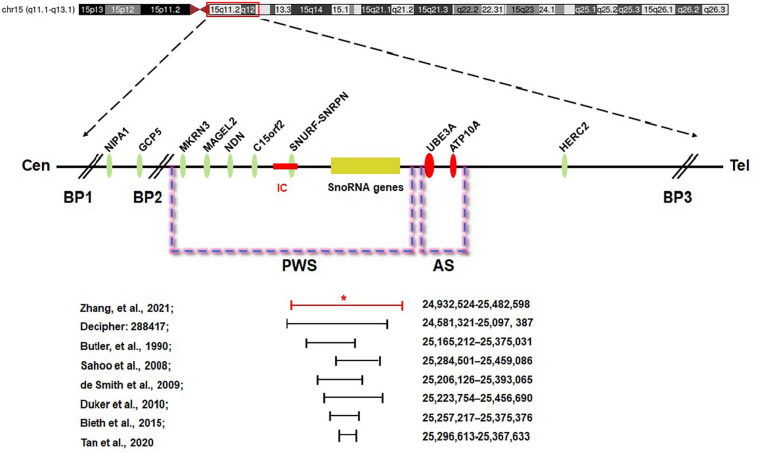
Prader–Willi syndrome (PWS) and Angelman syndrome (AS) domain in proximal chromosome 15q11-q13. The positions of genes (oval) in PWS, AS, and the IC are illustrated. CEN, centromere; Tel, telomere; IC, PWS imprinting center (red rectangle); BP, breakpoint; *the breakpoint of the proband.

The minimal critical region for PWS is proposed to be approximately 95 kb in size (at chr15:25280000-25375000, genome build hg 19) and contains two C/D box snoRNAs—the SNORD116 cluster and SNORD109A—as the only putative functional genes (Figure 5; Butler, 1990; Sahoo et al., 2008; de Smith et al., 2009; Duker et al., 2010; Bieth et al., 2015; Hassan and Butler, 2016; Tan et al., 2020). In review of the regions of deletion in the present and previously described cases that exhibit the key characteristics of the PWS phenotype, the SNORD116 cluster, SNORD109A, and the Imprinted in Prader–Willi (IPW) exons were found to be consistently deleted. In addition, by contrasting against PubMed, DECIPHER, and ClinVar database, the microdeletion in our patient was found to share a great similarity to the case reported in DECIPHER (patient number 288417). The deletion in patient 288417 of DECIPHER database, detected as 516 kb in size, encompassed five OMIM genes: *NPAP1*, *PWRN1*, *SNHG14*, *SNORD116*, and *SNRPN*. Also, patient 288417 showed a core phenotype characterized by obesity, aggressive behavior, intellectual disability, and psychosis (information of infant period was not provided), pointing toward a causative role of the genes in the minimal critical region in the broader phenotype of typical PWS. Furthermore, in animal models of PWS, knockout Snord116 mice displayed cognitive deficits (Adhikari et al., 2019), growth retardation (Ding et al., 2008), hyperphagia, and marked obesity (Polex-Wolf et al., 2018; Yang et al., 2019). So, all these findings indicate that PWS with microdeletion disrupting the IC should be considered in patients with hypotonia and developmental delay, even in the absence of the striking facial features. Furthermore, our research has provided further evidence that deletion of the SNORD116 region is sufficient to cause the key characteristics of PWS; therefore, suspicion of PWS should be aroused despite atypical physical features and rapid progression of the disorder. Meanwhile, the silent transmission of PWS IC microdeletion through the female germline has been recognized to be highly confounding for diagnostic testing and genetic counseling in affected patients and families. These results also suggest that other genes in the region may make specific phenotypic contributions, which necessitate further research and exploration to better understand the role of genes in the IC.

## Conclusion

Overall, the present study finds that the symptoms of our patient are distinct from, and may be more severe than, those of typical PWS cases. The familial occurrence and atypical clinical features were challenging to our diagnostic strategy. Based on the results of our study, PGD was applied successfully to exclude imprinting deficiency in preimplantation embryos before transfer into the mother’s uterus. As expected, the mother gave birth to a healthy boy. Our study may be especially instructive regarding accurate diagnosis, differential diagnosis, genetic counseling, treatment, and PGD for familial PWS patients.

## Data Availability Statement

The data presented in the study are deposited in the NODE BioProject repository, accession numbers are OEP001280 and OEP001281.

## Ethics Statement

The studies involving human participants were reviewed and approved by the Ethics Committee of Qilu Children’s Hospital of Shandong University. Written informed consent to participate in this study was provided by the participants’ legal guardian/next of kin. Written informed consent was obtained from the individual(s), and minor(s)’ legal guardian/next of kin, for the publication of any potentially identifiable images or data included in this article.

## Author Contributions

YLiu, YY, and SZ supervised the project. KZ and SL wrote the manuscript. WG, KZ, YLiu, and YLv were involved in the clinical diagnosis and whole-exome sequencing and bioinformatics analysis. DW and MG performed the karyotype analysis and chromosome microarray analysis. XL and ZG participated in case follow-up. All authors were involved in the conception, experiment design, and data analysis and approved the final manuscript.

## Conflict of Interest

The authors declare that the research was conducted in the absence of any commercial or financial relationships that could be construed as a potential conflict of interest.

## Publisher’s Note

All claims expressed in this article are solely those of the authors and do not necessarily represent those of their affiliated organizations, or those of the publisher, the editors and the reviewers. Any product that may be evaluated in this article, or claim that may be made by its manufacturer, is not guaranteed or endorsed by the publisher.

## Footnotes

1
http://bio-bwa.sourceforge.net/

2
www.broadinstitute.org/gatk

3
annovar.openbioinformatics.org/en/latest/

4
http://www.ncbi.nlm.nih.gov/omim

5
http://decipher.sanger.ac.uk/

6
http://projects.tcag.ca/variation

7
http://dbsearch.clinicalgenome.org/search/

## Supplementary Material

Supplementary Table 1 The variants identified in WES for the family.
