# Supplementary material for: Transmission of a Novel Imprinting Center Deletion Associated With Prader–Willi Syndrome Through Three Generations of a Chinese Family: Case Presentation, Differential Diagnosis, and a Lesson Worth Thinking About
Source: Front Genet. 2021 Aug 24;12:630650. doi: 10.3389/fgene.2021.630650 (PMC8421676; doi:10.3389/fgene.2021.630650)
Supplement: Supplementary Table 1 — The variants identified in WES for the family. [file Table_1.DOCX]

| Gene | Location in chromosome | Change in nucleotide | NM_NO. | Gene subregion | Homozygous/  Heterozygote | Amino acid Change | Pathogenicity | Disease/  phenotype | Genetic mode |  | Variation source |
| --- | --- | --- | --- | --- | --- | --- | --- | --- | --- | --- | --- |
| \| *GBA* \|  \|  \|  \|  \|  \|  \|  \|  \|  \|  \| \| --- \| --- \| --- \| --- \| --- \| --- \| --- \| --- \| --- \| --- \| --- \| | Chr1:155205620 | c.1240C>G | NM_001005741.2 | CDS9 | Hetero | p.V414L | Uncertain | Gaucher disease | AR |  | Paternal |
| *SLC12A3* | Chr16:56904571 | c.775G>A | NM_000339.2 | CDS6 | Hetero | p.D259N | Uncertain | Gitelman Syndrome | AR |  | Paternal |
| *PNPT1* | Chr2:55887294 | c.1282C>T | NM_033109.3 | CDS15 | Hetero | p.E428K | Uncertain | 1. Combined oxidative phosphorylation deficiency 13  2. Deafness 70 | 1.AR  2.AR |  | Paternal |
| *BCL11A* | Chr2:60688455 | c.1592C>T | NM_022893.3 | CDS4 | Hetero | p.G531D | Uncertain | Dias-Logan syndrome | AD |  | Paternal |
| *CEP135* | Chr4:56886856 | c.3230G>A | NM_025009.4 | CDS23 | Hetero | p.R1077Q | Uncertain | Primary microcephaly 8 | AR |  | Paternal |
| *TRAPPC9* | Chr8:140743419 | c.3626G>A | NM_031466.5 | CDS23 | Hetero | p.T1209M | Uncertain | Mental retardation 13 | AR |  | Maternal |
| *DOLK* | Chr9:131708766 | c.817G>A | NM_014908.3 | CDS1 | Hetero | p.P273S | Uncertain | Congenital disorder of glycosylation, type Im | AR |  | Maternal |
| *ANK3* | Chr10:61833869 | c.6770G>A | NM_020987.3 | CDS37 | Hetero | p.P2257L | Uncertain | Mental retardation 37 | AR |  | Paternal |
| *SCN8A* | Chr12:52139741 | c.2053A>G | NM_014191.3 | CDS12 | Hetero | p.M685V | Uncertain | 1. Cognitive impairment with or without cerebellar ataxia  2. Developmental and epileptic encephalopathy 13  3. Seizures, benign familial infantile, 5 | 1.AD  2.AD  3.AD |  | Maternal |
| *COQ9* | Chr16:57486748 | c.278A>G | NM_020312.3 | CDS3 | Hetero | p.Y93C | Uncertain | Primary coenzyme Q10 deficiency 5 | AR |  | Maternal |
| *SHROOM4* | ChrX:50345760 | c.3815G>A | NM_020717.3 | CDS7 | Hetero | p.S1272F | Uncertain | Stocco dos Santos X-linked mental retardation syndrome | XL |  | Maternal |

AD: Autosomal dominant; AR: Autosomal recessive; XL: X chromosome linkage.

**SUPPLEMENTS:**

Table S1. The variants identified in WES for the family
